# A mechanism for the sharp transition of morphogen gradient interpretation in *Xenopus*

**DOI:** 10.1186/1471-213X-7-47

**Published:** 2007-05-16

**Authors:** Yasushi Saka, James C Smith

**Affiliations:** 1 Wellcome Trust/Cancer Research UK Gurdon Institute and Department of Zoology, University of Cambridge, Tennis Court Road, Cambridge CB2 1QN, UK; 2 Interdisciplinary Research Institute, Institut de Biologie de Lille, 1 rue du Professeur Calmette, BP447, 59021 Lille Cedex, France

## Abstract

**Background:**

One way in which positional information is established during embryonic development is through the graded distribution of diffusible morphogens. Unfortunately, little is known about how cells interpret different concentrations of morphogen to activate different genes or how thresholds are generated in a morphogen gradient.

**Results:**

Here we show that the concentration-dependent induction of the T-box transcription factor *Brachyury *(*Xbra*) and the homeobox-containing gene *Goosecoid *(Gsc) by activin in *Xenopus *can be explained by the dynamics of a simple network consisting of three elements with a mutual negative feedback motif that can function to convert a graded signal (activin) into a binary output (*Xbra *on and *Gsc *off, or vice versa). Importantly, such a system can display sharp thresholds. Consistent with the predictions of our model, *Xenopus *ectodermal cells display a binary response at the single cell level after treatment with activin.

**Conclusion:**

This kind of simple network with mutual negative feedback might provide a general mechanism for selective gene activation in response to different levels of a single external signal. It provides a mechanism by which a sharp boundary might be created between domains of different cell types in response to a morphogen gradient.

## Background

One way in which positional information might be established during embryonic development is through the graded distribution of diffusible morphogens, including members of the TGF-β, FGF and Wnt families of growth factors [1-3]. Although progress is being made in coming to understand the ways in which morphogens can traverse fields of cells [4-6], rather little is known about how cells interpret different concentrations of morphogen to activate different genes or how thresholds are generated in a morphogen gradient. Recent studies indicate that morphogens frequently exert their effects through the post-translational activation of a single transcription factor, which in turn induces downstream target genes in a concentration-dependent manner. For example, dorso-ventral patterning in *Drosophila *embryo is controlled by the graded activation of the NF-kB-like transcription factor Dorsal [7].

In *Xenopus*, members of the TGF-β family such as activin and the nodal-related proteins act as morphogens and are essential for mesoderm formation [2,8]. They activate downstream gene expression in a concentration-dependent manner, with low concentrations activating the T-box transcription factor *Brachyury *(*Xbra*) and high concentrations inducing the homeobox-containing gene *Goosecoid *(*Gsc*) [9]. Changes in the extracellular activin concentration are reflected by differences in the concentration of nuclear Smad2, the effector of activin signalling [10]. But how do differences in effector concentration cause the activation of different target genes? Several mechanisms to explain this phenomenon have been proposed [1], but it is not clear how they generate sharp thresholds, with small differences in morphogen concentration yielding qualitative differences in gene expression.

In this paper we show that the concentration-dependent induction of *Xbra *and *Gsc *by activin can be explained by the dynamics of a mutual negative feedback motif that can be adapted to function as a module to convert a graded signal (activin) into a binary output (*Xbra *on and *Gsc *off, or vice versa). We note that this system can display sharp thresholds, and it provides a plausible mechanism by which cells might distinguish between small concentration differences in a morphogen gradient and thereby create a boundary between two different cell types. Consistent with the predictions of our model, *Xenopus *ectodermal cells display a binary response at the single cell level after treatment with activin. We suggest that this mutual negative feedback gene network represents a general mechanism for selective gene activation in response to different levels of a single external signal.

## Results

### A mutual negative feedback motif can explain the concentration-dependent induction of *Xbra *and *Gsc*

**Figure 1:**
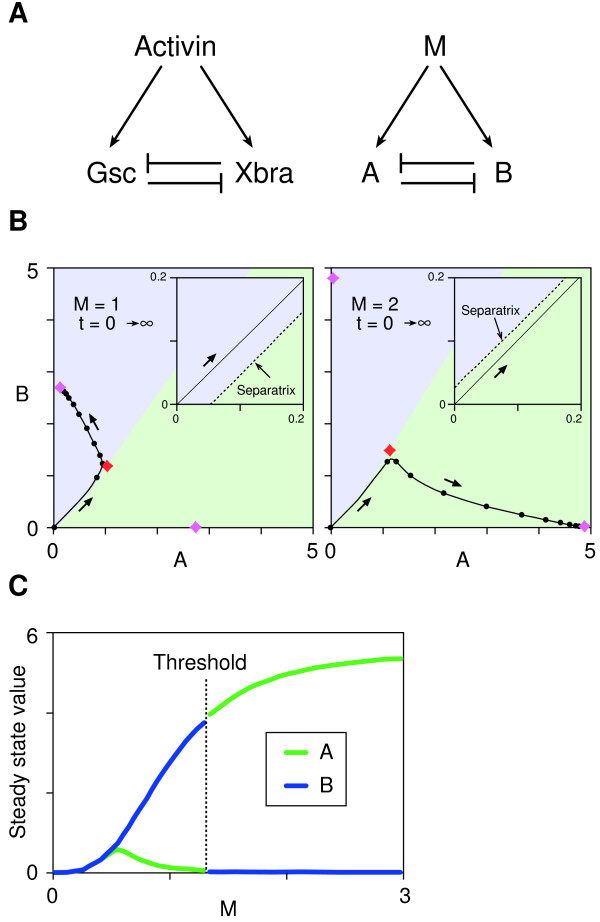
**Bifurcation and thresholds in a simple network with a mutual negative feedback motif**. (A) Activin, *Xbra *and *Gsc *form a network in which *Xbra *and *Gsc *are both induced by activin and can inhibit each other's expression. This network can be abstracted into a general network consisting of M, A and B as illustrated. (B) Trajectory of the phase point in the phase plane (A, B). The behaviours of the network fall into three categories (see Additional file 1). These panels show a typical example of bifurcation with a threshold. The phase plane is divided into two basins (blue and green) by a border (separatrix) and each basin has one stable point (node). The phase point moves as indicated by the arrows. Movement of the phase point is analogous to a ball rolling in a landscape (the phase plane), which features a summit (saddle point: red diamond) and a ridge (separatrix). These features cause the ball to roll down into one low point or the other (nodes: purple diamonds) in each basin. The position of the separatrix depends on the value of M, with the initial phase point (0, 0) in the blue basin when M is below the threshold (left panel) or in the green basin when M is above the threshold (right panel). Insets show magnified views of the initial point. Black dots indicate the position of the phase point with interval of t = 0.5. Purple diamond, nodes; Red diamond, saddle point. (C) Steady state values of (A, B) are plotted against M. The threshold is between M = 1 and M = 2. The parameter values used for the simulation are k_a _= 5.5, k_b _= 5.4, α = 6, β = 3, kd_a _= kd_b _= 1.

During *Xenopus *development, activin and the nodal-related proteins activate gene expression in a concentration-dependent manner, with low concentrations of activin inducing the expression of *Xbra *while high concentrations activate *Gsc *in isolated *Xenopus *animal pole regions. These two genes repress each other's expression, thereby creating a regulatory network based on mutual negative feedback: Gsc can repress transcription of *Xbra *by binding directly to its promoter [11,12], while Xbra suppresses *Gsc *by inducing the expression of *Xom*, a repressor of *Gsc *(also known as *Xvent2*, *Vox *and *Tbr-1*), [13-17] (Fig. 1A, left panel).

We have attempted to predict the concentration-dependent effects of activin, and the dynamic aspects of its inductive activity, from this network structure. Our model consists of three interacting elements, M, A and B (Fig. 1A, right panel). M induces both A and B, while A suppresses the expression of B and vice versa. The dynamics of the network are described by two time-dependent ordinary differential equations:

dAdt=ka1+Bβ⋅Mμ1+Mμ−kda⋅A
 MathType@MTEF@5@5@+=feaafiart1ev1aaatCvAUfKttLearuWrP9MDH5MBPbIqV92AaeXatLxBI9gBaebbnrfifHhDYfgasaacH8akY=wiFfYdH8Gipec8Eeeu0xXdbba9frFj0=OqFfea0dXdd9vqai=hGuQ8kuc9pgc9s8qqaq=dirpe0xb9q8qiLsFr0=vr0=vr0dc8meaabaqaciaacaGaaeqabaqabeGadaaakeaadaWcaaqaaiabdsgaKjabdgeabbqaaiabdsgaKjabdsha0baacqGH9aqpdaWcaaqaaiabdUgaRnaaBaaaleaacqWGHbqyaeqaaaGcbaGaeGymaeJaey4kaSIaemOqai0aaWbaaSqabeaaiiGacqWFYoGyaaaaaOGaeyyXIC9aaSaaaeaacqWGnbqtdaahaaWcbeqaaiab=X7aTbaaaOqaaiabigdaXiabgUcaRiabd2eannaaCaaaleqabaGae8hVd0gaaaaakiabgkHiTiabdUgaRjabdsgaKnaaBaaaleaacqWGHbqyaeqaaOGaeyyXICTaemyqaeeaaa@4D43@

dBdt=kb1+Aα⋅Mμ1+Mμ−kdb⋅B
 MathType@MTEF@5@5@+=feaafiart1ev1aaatCvAUfKttLearuWrP9MDH5MBPbIqV92AaeXatLxBI9gBaebbnrfifHhDYfgasaacH8akY=wiFfYdH8Gipec8Eeeu0xXdbba9frFj0=OqFfea0dXdd9vqai=hGuQ8kuc9pgc9s8qqaq=dirpe0xb9q8qiLsFr0=vr0=vr0dc8meaabaqaciaacaGaaeqabaqabeGadaaakeaadaWcaaqaaiabdsgaKjabdkeacbqaaiabdsgaKjabdsha0baacqGH9aqpdaWcaaqaaiabdUgaRnaaBaaaleaacqWGIbGyaeqaaaGcbaGaeGymaeJaey4kaSIaemyqae0aaWbaaSqabeaaiiGacqWFXoqyaaaaaOGaeyyXIC9aaSaaaeaacqWGnbqtdaahaaWcbeqaaiab=X7aTbaaaOqaaiabigdaXiabgUcaRiabd2eannaaCaaaleqabaGae8hVd0gaaaaakiabgkHiTiabdUgaRjabdsgaKnaaBaaaleaacqWGIbGyaeqaaOGaeyyXICTaemOqaieaaa@4D47@

M, A and B are the concentrations of each element, k_a _and k_b _are the synthesis rates (or maximum flux rates of A and B into a specific compartment of the system such as the nucleus), and kd_a _and kd_b _are constants describing the decay (or irreversible inactivation such as protein degradation) of A and B, respectively. α and β are the cooperativities of repression by A of B and by B of A respectively, and μ is the cooperativity of induction by M. These cooperativities (or Hill coefficients) introduce non-linearity within the network and are important for its bistability [18,19]. The cooperativity of induction (μ) is set to be the same for A and B, and kd_a _and kd_b _are set to unity. Neither of these simplifications affects the overall behaviour of the system described in Additional file 1. M corresponds to the concentration of activin. The initial condition (at t = 0) for the simulations is (A, B) = (0, 0), reflecting the fact that expression of both *Xbra *and *Gsc *is induced by activin.

For the sake of simplicity we assume that M stays constant throughout the simulation. A few observations justify this assumption. First, cells respond by a 'ratchet mechanism' to the highest concentration of activin they are exposed to during their period of competence, and the timing of the response is related to the developmental stage of the cells, and not to the time of first exposure to activin [2]. Second, the response of cells to activin is proportional to the absolute number of bound receptors that are internalised by the endocytic pathway and these remain active for several hours after a brief exposure of cells to activin [20]. We note that the model described by equations (1) and (2) is a modified form of the dimensionless 'toggle model' [21] or 'repressor-repressor switch' [18].

**Figure 2:**
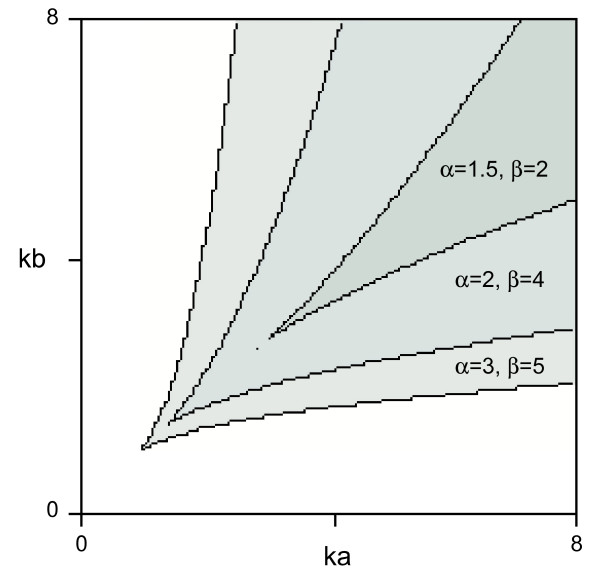
**A plot of the area in the parameter plane (k_a_, k_b_) that allows the system to bifurcate**. Bistability requires the balanced rates of synthesis of A and B (k_a _and k_b_). The product of cooperativity of the mutual repression between A and B must be greater than 1 (i.e. αβ > 1) for the system to bifurcate. μ = 3, kd_a _= kd_b _= 1.

Depending on the parameter values, the behaviour of the network illustrated in Fig. 1A can be classified as monostable or bistable (see Additional file 1). Bistability (i.e. the condition under which two stable states exist) requires the balanced rates of synthesis of A and B (k_a _and k_b_) (Fig. 2). Our result is consistent with previous studies showing that the product of cooperativity of the mutual repression between A and B must be greater than 1 (i.e. αβ >1) [18,21], reflecting the fact that our model and theirs both have a mutual negative feedback motif at their cores. Most significantly, when certain conditions are met, the system reaches one of two opposing stable states (high A and low B, or vice versa) depending on the value of M (Fig. 1B), with bifurcation of the system occurring with a sharp threshold (Fig. 1C, see also Additional file 1). This behaviour resembles the concentration-dependent effects of a morphogen. The choice between the two opposing steady states with a given value of M depends on the parameter values and the initial conditions.

**Figure 3:**
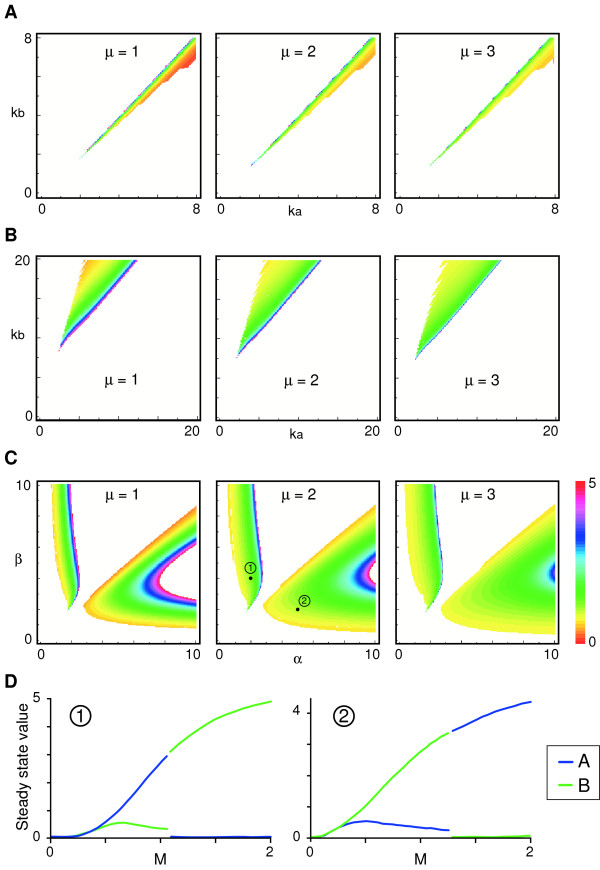
**Relationship between parameters and threshold values of M**. Threshold values are calculated and plotted and colour-coded as indicated. White areas indicate either that a threshold does not exist or that its value is above 5. (A) Threshold values are plotted in parameter plane (k_a_, k_b_). α = 6, β = 3, kd_a _= kd_b _= 1. (B) Setting different values for the decay constants kd_a _and kd_b _significantly broadens the range of possible parameter values of k_a _and k_b _that allows a threshold generation. Degradation constants are set to kd_a _= 1 and kd_b _= 5. Note that the area in the parameter plane (k_a_, k_b_) that allows threshold (coloured area) is shifted and becomes much broader compared to (A). α = 6, β = 3. (C) Threshold values are plotted in parameter plane (α, β). k_a _= 5.5, k_b _= 5.4. (D) Steady state values of A and B plotted against M at the black dots in (C). Left panel, (α, β) = (2, 4). Right panel, (α, β) = (5, 2). k_a _= 5.5, k_b _= 5.4, kd_a _= kd_b _= 1. Note that the two areas in (C) where the system is bistable with a threshold shows opposite steady state profiles. When α < β (area 1 in the middle panel in C), A is on and B is off with low M, and vice versa with high M at steady state. When α > β (area 2 in the middle panel in C), B is on and A is off with low M, and vice versa with high M at steady state.

**Figure 4:**
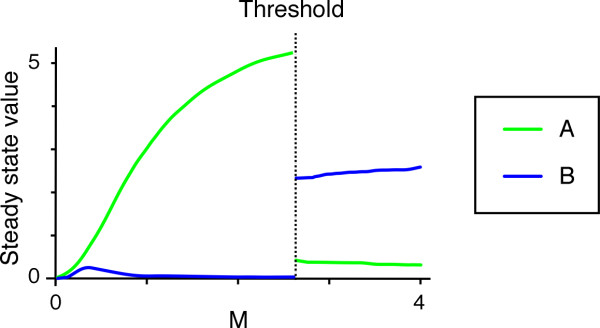
**Typical example of threshold creation when cooperativities of repression α and β are equal**. Steady state values of A and B are plotted against M when α = β = 3, (k_a_, k_b_) = (6, 14) and (kd_a_, kd_b_) = (1, 5), μ = 2.

We have examined the conditions under which a threshold response can occur. Firstly, bistability is necessary but not sufficient for threshold formation (see Additional file 1). Secondly, the synthesis rates k_a _and k_b _must be well balanced for threshold formation to occur and the parameter choice here is more limited than for bistability (Fig. 3A, compare with Fig. 2). Thirdly, when the values of decay constants kd_a _and kd_b _are different, the range of parameter choice for synthesis rates k_a _and k_b _becomes significantly larger and threshold values become more robust to parameter fluctuations (Fig. 3B). It also seems that, with a larger value of cooperativity of induction μ, threshold values are less sensitive to fluctuations of the other parameters (Fig. 3A–C). Interestingly, the establishment of the threshold proves to be rather insensitive to small changes of the parameters for cooperativity of repression α and β (Fig. 3C). Finally, the numerical simulations indicate that, if α = β and decay constants kd_a _= kd_b _at the same time, a threshold cannot be established with any pair of values of synthesis rates k_a _and k_b _(Fig. 3C and data not shown). However, even if α = β, a threshold can be formed when appropriate parameter values of (k_a_, k_b_) and (kd_a_, kd_b_) are chosen (Fig. 4).

The simulations also indicate that if α = β, the parameters must satisfy k_a _> k_b _and kd_a _> kd_b_, or k_a _< k_b _and kd_a _< kd_b _in order to create a threshold (Fig. 4 and data not shown). When k_a _< k_b _and kd_a _< kd_b_, it seems always to be the case that A is on and B is off with low M at steady state, and vice versa with high M (Fig. 4 is an example). This principle of the system's behaviour might be explained as follows: at low M, the decay rate predominates over the synthesis rate, so that A, which has a lower decay rate, comes to be expressed at the expense of B. At high M, rates of synthesis dictate the behaviour of the system and B, with its higher synthesis rate, is eventually expressed at the expense of A.

**Figure 5:**
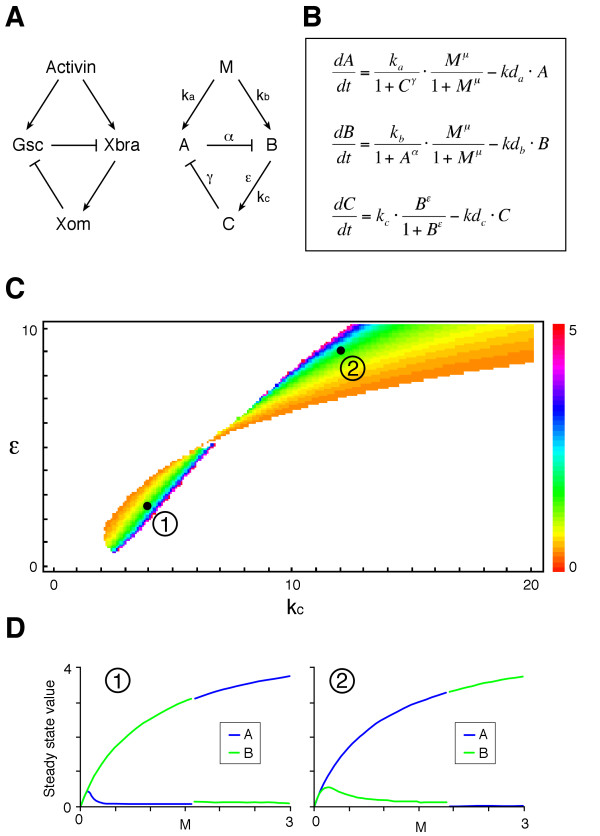
**A mutually repressive network with an additional element**. (A) The homeobox-containing transcription factor Xom mediates the repression of *Gsc *by Xbra. The gene network can be abstracted into a general network consisting of M, A, B and C as illustrated. (B) Ordinary differential equations describing the dynamics of the network in (A). k_a_, k_b _and k_c _are respectively the rates of synthesis of A, B and C. α and γ are the cooperativities of repression by A and C, ε and μ are the cooperativities of induction by B and M, respectively. kd_a_, kd_b _and kd_c _are the decay rate constants. The dynamics of A and B are similar to those in Additional file 1 and show bistability. (C) Simulations were performed with k_a _= k_b _= 5 and α = γ = 3 (μ = 1, kd_a _= kd_b _= kd_c _= 1). Threshold values of M, which are colour-coded, are plotted in the parameter plane (k_c_, ε). k_c _and ε determine how the value of C changes over time. There are two areas in the parameter plane where the system is bistable with a threshold. With smaller values of k_c _and ε (area 1), B is on and A is off with low M, and vice versa with high M at steady state. With larger values of k_c _and ε (area 2), A is on and B is off with low M, and vice versa with high M at steady state. (D) Typical examples (black dots in (C)) of steady state values of A and B plotted against M. Left panel, (k_c_, ε) = (4, 2.5). Right panel, (k_c_, ε) = (12, 9). If smaller values of k_c _and ε are favoured in nature, the above observation may explain why at steady state and with low activin Xbra (which corresponds to B) is on and Gsc (which corresponds to A) is off, and vice versa with high activin.

The simulation in Fig. 1B shows the induction of A and B at two levels of M, one which results in expression of A at steady state and one which results in expression of B. Importantly, in both cases, the immediate response to M before steady state is to activate both A and B, and this behaviour recapitulates the expression dynamics of *Xbra *and *Gsc *in *Xenopus *animal pole regions after treatment with activin [22-24]. Incorporation of the transcription factor Xom in the simulation by adding an extra component in the scheme illustrated in Fig. 1A does not change the fundamental behaviour of the system and the dynamics of A and B can be categorised into three patterns: monostable, bistable without threshold, or bistable with threshold (see Additional file 1). This is presumably because the mutual negative relations between A and B stay the same. By choosing a set of appropriate parameters, it shows bistability with a threshold (Fig. 5).

### Mutual exclusion of *Xbra *and *Gsc *expression at the single cell level

**Figure 6:**
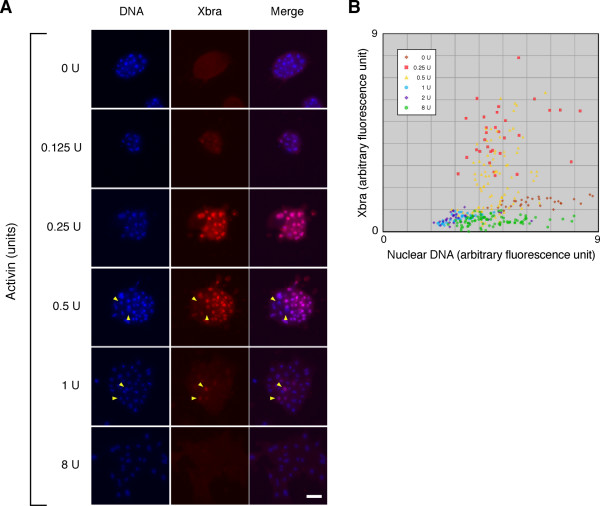
**Heterogeneous expression of Xbra in *Xenopus *animal pole blastomeres**. (A) Heterogeneous expression of Xbra in *Xenopus *animal pole blastomeres treated with uniform concentrations of activin. Dissociated *Xenopus *animal cap cells were treated with various concentrations of activin as indicated. The cells were fixed and stained by indirect immunofluorescence using an anti-Xbra antibody. Yellow arrowheads indicate the nuclei that are Xbra-negative among positive nuclei (0.5 U) or vice versa (1 U). Cells were counterstained with Hoechst33342 (DNA). Scale bar = 50 μm. (B) Plot of Xbra versus DNA fluorescences. The fluorescence was quantified as described in Methods.

To ask whether the thresholds predicted by the simulation can exist at the single cell level in vivo, dissociated *Xenopus *animal pole cells were treated with different concentration of activin and cultured on fibronectin-coated glass. Cells were fixed after 7 hours and observed by indirect immunofluorescence microscopy using an anti-Xbra antibody. As described previously, *Xbra *was activated in a concentration-dependent manner, with maximal expression at 0.25 and 0.5 U/ml activin (Fig. 6). Interestingly, cells did not respond to activin uniformly; even at 0.5 U/ml some cells did not express *Xbra *(Fig. 6A, yellow arrowheads and 6B). The origin of this heterogeneity is unknown, although it may be due in part to the bifurcation properties of the system illustrated in Fig. 1 and also to the intrinsic stochastic nature of gene expression [25]. In particular, the border (separatrix) that divides the phase plane is close to the point of the initial state (A = B = 0) when the value of M is near the threshold value (Fig. 1B). This would make the system sensitive to noise which, in a real *in vivo *system such as *Xenopus *animal cap cells, might influence the choice between the two stable states. Thus, two populations of cells (with high *Xbra *and low *Gsc *expression, or vice versa) would be produced when the concentration of activin is close to the threshold level. In fact, quantification of levels of fluorescence show that the expression level of Xbra is somewhat heterogeneous at both 0.25 and 0.5 U/ml activin, but much more so at 0.5 U/ml (Fig. 6B). It is likely that the higher of the two concentrations is close to the threshold at which Xbra expression is extinguished and Gsc is activated. The population of cells might therefore be a mixture of 'Xbra on' and 'Xbra off'. The lower concentration of 0.25 U/ml is likely to cause all cells to activate Xbra, and the lower level of heterogeneity observed at this concentration is likely to reflect the stochastic nature of gene expression.

**Figure 7:**
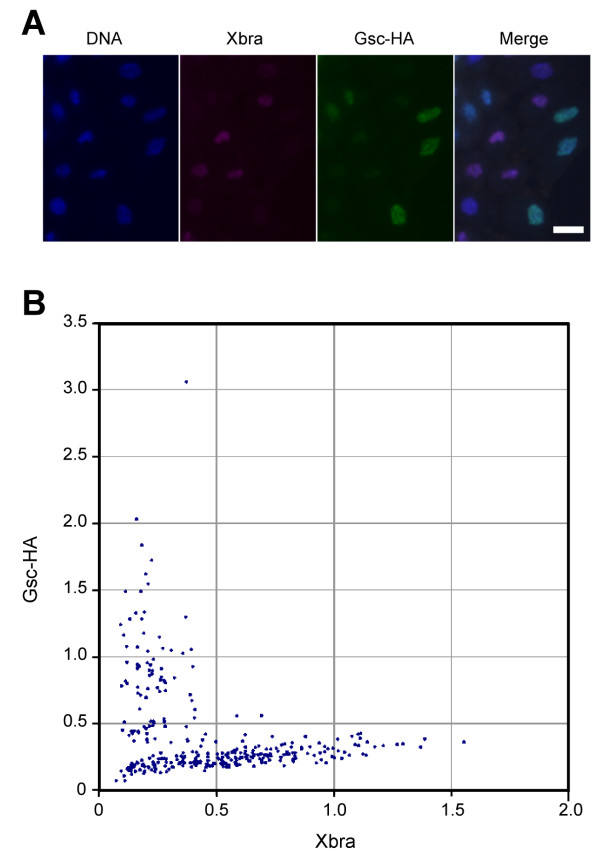
**Mutually exclusive expressions of Xbra and Gsc in *Xenopus *animal pole blastomeres**. (C) Mutually exclusive expressions of Xbra and Gsc in *Xenopus *animal pole blastomeres. Fertilised *Xenopus *embryos were injected with RNA encoding HA-tagged Gsc (Gsc-HA). Animal pole regions derived from these injected embryos and from uninjected embryos were mixed, dissociated and treated with 0.5 U/ml of activin. They were fixed after 7 hours of culture and stained with Hoechst 33342 (DNA) and processed for anti-Xbra and anti-HA staining. Scale bar = 20 μm. (D) Arbitrary fluorescence levels of anti-Xbra and anti-HA (Gsc) staining shown in (C) were calculated as described in Methods.

The results described above suggest that the mutual exclusion of *Xbra *and *Gsc *expression occurs at the single cell level. To test this idea, we injected RNA encoding HA-tagged *Gsc *into *Xenopus *embryos at the one-cell stage and cultured these embryos to mid-blastula stage 9. Animal pole blastomeres derived from such embryos were mixed with animal pole cells from uninjected embryos, and they were then dissociated and cultured for 7 hours in the presence of 0.5 U/ml activin, after which Xbra and Gsc-HA were detected by indirect immunofluorescence. *Xbra *proved to be activated only in cells that expressed the lowest levels of Gsc-HA (Fig. 7A), a conclusion that was confirmed by measuring the levels of Xbra and Gsc-HA in each cell and plotting them against each other (Fig. 7B).

## Discussion

The results described in this paper show that the concentration-dependent induction of *Xbra *and *Gsc *by activin can be explained by the dynamics of a simple network with only three elements (Fig. 1A), in which a mutual negative feedback motif converts a graded signal (activin) into a binary output (*Xbra *on and *Gsc *off, or vice versa). The behaviour of mutual negative feedback motifs of this sort has been examined theoretically [18,26] and experimentally [21], and it may provide a general mechanism for selective gene activation in response to different levels of a single external signal. Indeed, mutual negative feedback motifs play crucial roles in the behaviour of several biological processes, including cell type specification in the vertebrate neural tube [27,28], embryonic segmentation [29] and photoreceptor cell fate decision in *Drosophila *[30]. However, to our knowledge, the dynamic behaviour of such a mutual negative feedback motif to a single signal as illustrated in Fig. 1A has not previously been investigated. The results described in this paper demonstrate that such a system can display sharp thresholds. Although the now widely-accepted concept of a morphogen gradient requires threshold behaviours of this sort, rather few suggestions as to how thresholds are generated have so far been made [31,32]. Our model provides a plausible mechanism by which a sharp boundary might be created between domains of different cell types in response to a morphogen gradient. It also explains how cells interpret a small concentration change in a morphogen gradient.

Our model builds on previous work [31,32], and especially on experiments in *Drosophila *showing that different promoter affinities might underlie differential responses to the Bicoid morphogen gradient [33,34]. In our model, promoter affinities and strengths are incorporated into the parameters of synthesis rates k_a _and k_b_. It seems that balanced but not necessarily equal rates of synthesis are required for threshold formation, but equally important are the stabilities of A and B, as represented by the decay rates kd_a _and kd_b_. Indeed, the system becomes more robust when kd_a _> kd_b _(or vice versa) because there are more choices of parameter pairs for synthesis (k_a_, k_b_) that permit threshold generation (compare Fig. 3B and 3C). It also means that thresholds become less sensitive to gene expression noise if the decay rates are different. Recent evidence suggests that pre-steady state interpretation of the Bicoid morphogen gradient contributes to the robustness of the system and ensures the accurate expression patterns of target genes [35]. Although the morphogen level (M) stays the same in our model simulation, similar dynamic interpretations of changes in activin concentration might also provide robustness to threshold determination in our model. Our results also indicate that k_a _< k_b _and kd_a _< kd_b _(or vice versa) must be satisfied in order to create a threshold if the other parameters such as the cooperativities of repression are the same. In principle these predictions might be tested by manipulating the promoter strengths of *Xbra *and *Gsc *together with the stabilities of their gene products, but such experiments would be technically challenging. Such attempts have also been hampered by the unavailability of anti-Gsc antisera.

Our model simulation recapitulates the induction and the co-expression of *Xbra *and *Gsc *immediately after exposure to activin, which cannot be deduced solely by the mutual repression between *Xbra *and *Gsc*. We note that the expression domains of *Xbra *and *Gsc *overlap at the early gastrula stage but then resolve by the end of gastrulation [11]. And, also consistent with the model, the down regulation of *Xbra *in response to high concentrations of activin requires protein synthesis [23], indicating that mutual inhibition plays an essential role in this process. Although transient, the ubiquitous expression of *Xbra *in mesoderm is essential for morphogenetic movements during gastrulation [36,37]. This illustrates the importance of the dynamic behaviour of a gene regulatory network as well as its steady state outcome, especially in the context of a dynamic process such as animal development.

Interestingly, we find that exposure to intermediate concentrations of activin causes different cells to make opposite binary decisions (*Xbra *on or off; Fig. 6). A similar heterogeneity in *Xbra *expression was found in single marginal zone cells of the *Xenopus *embryo at the early gastrula stage [24]. This observation points to the importance of cell-cell communication in refining the borders between populations of different cell types created by a morphogen gradient. This communication may involve a community effect [38], perhaps requiring positive feedback between *Xbra *and FGF signalling [39]. Lack of this positive feedback in dissociated *Xenopus *animal pole cells may also contribute to the observed heterogeneity in Xbra expression. It should be possible to incorporate such feedback to refine our model.

It is also possible, by combining additional feedback events, to create multiple thresholds in response to a morphogen gradient. For example, during neuronal cell fate specification in the ventral neural tube, 'class I' and 'class II' homeodomain transcription factors, which are regulated by the morphogen Sonic Hedgehog, inhibit each other's expression [27,28]. This sort of combinatorial mechanism may be made more general (see Additional file 2).

## Conclusion

Our simulation of a simple network of three elements with a mutual negative feedback motif recapitulates the dynamics of the concentration-dependent induction of *Xbra *and *Gsc *by activin in *Xenopus*. Our model provides a mechanism by which a sharp boundary might be created between domains of different cell types in response to a morphogen gradient.

## Methods

### Numerical simulations and calculations

Numerical simulations and calculations were performed using Mathematica (Ver 5.2, Wolfram Research). The package of functions and programs used is available upon request. Part of the algorithm was adopted from Murrell [40].

### *Xenopus *embryo manipulation and microinjection

Fertilisation and culture of *Xenopus *embryos were performed as described [23] and recombinant human activin A was obtained as described [41]. RNA injection was performed according to Smith [42]. Animal pole regions were dissected from blastulae and cells were dissociated in calcium- and magnesium-free medium (Tris 75 mM pH7.5, NaCl 880 mM, KCl 10 mM, NaHCO_3 _24 mM) for 30–45 minutes at room temperature before treating with activin. *Gsc *tagged with an HA epitope at its C-terminus, cloned in the vector pCS2+, was transcribed in vitro using mMESSAGE mMACHINE (Ambion).

For the experiment shown in Fig. 7, fertilised *Xenopus *embryos were injected with 200 pg RNA encoding HA-tagged Gsc (Gsc-HA). Animal pole regions derived from these injected embryos and from uninjected embryos were mixed in a ratio of 5:1. Blastomeres derived from this mixed population of animal pole regions were dissociated and treated with 0.5 U/ml of activin. They were fixed after 7 hours of culture and stained with Hoechst 33342 and processed for anti-Xbra and anti-HA staining. Dissociated animal cap cells were cultured in 75% Normal Amphibian Medium [43] containing 0.1% bovine serum albumin (Sigma, Fraction V, A9647) in glass-bottomed microwell dishes (MatTek Co. P35GC-1.5-14-C) that had been covered overnight with a 0.002% solution of fibronectin (Sigma, F0895) and washed once with water before use.

### Indirect immunofluorescence microscopy and quantification of fluorescence

Cells were fixed in MEMFA [42] containing 1 mM EDTA and incubated with anti-Xbra [44] (1:1000) and anti-HA (1:10,000, clone 3F10 high affinity, Roche) antibodies. Secondary antibodies were Cy3-conjugated goat anti-rabbit IgG (H+L) (1.5 μg/ml, Jackson ImmunoResearch Laboratories) and Alexa488-conjugated anti-mouse IgG (2 μg/ml, Molecular Probes). Hoechst33342 (0.5 μg/ml, Molecular Probes) was used to visualize nuclear DNA. Images were acquired with a digital CCD camera (Hamamatsu C4742-95 Orca100) mounted on Leica DMIRB microscope, using Openlab software (Improvision). Quantification of fluorescence was performed using ImageJ [45]. Arbitrary fluorescence figures in Fig 7 were defined by the mean grey value of anti-Xbra (or anti-HA) fluorescence divided by mean grey value of DNA fluorescence in a region of interest (a nucleus).

## Authors' contributions

YS conceived and carried out the studies, and drafted the manuscript. JCS participated in the design of the study and helped to draft the manuscript. Both authors read and approved the final manuscript.

## Supplementary Material

Additional File 1 **Three possible behaviours for the system described in **Figure 1A: **monostable, bistable without a threshold, and bistable with a threshold**. This system has two variables (A and B) that change over time according to equations (1) and (2) in the main text. The state of the system at a given time point is defined by the values (A, B) and can be plotted as a single point (i.e. phase point) on the two-dimensional phase plane illustrated in Fig. 1B. The phase point moves around the phase space with a speed defined by the equations (1) and (2) in the main text. This system like others with a mutual negative feedback motif undergoes a saddle-node bifurcation (Kobayashi H et al (2004) *Proc Natl Acad Sci USA ***101: **8414–8419). Nodes and saddles are the points on the phase plane where the speed of the phase point is zero, and therefore derive from the solution of a coupled equation where the left hand sides of equations (1) and (2) are replaced with 0. In our system, there are three fixed points when bifurcation occurs, two of which are stable nodes and the other is an unstable saddle point. A saddle point is analogous to a pointed summit where a ball never rests. At steady state, the phase point rests at one of the two stable nodes (see Fig. 1B). The figure shows how the positions of these nodes and the saddle change in the phase plane (A, B) as M increases from 0 to 8 as indicated by the arrows. Green curves indicate the nodes where the phase point reaches at steady state after starting from (0, 0). (A) Monostable. There is just one node irrespective of the value of M. (B) Bistable without threshold. When M is small there is only one node. As M increases, bifurcation occurs (red triangle) and another node appears, but the phase point always ends up at the original node at steady state. (C) Bistable with a threshold. When M reaches the threshold (yellow diamond), the position of the phase point at steady state shifts from the original node to the other node created at the bifurcation. Bistability is necessary but not sufficient for a threshold to be generated. Click here for file

Additional File 2 **Creation of multiple thresholds by combination of mutual negative feedback motifs**. (A) A network structure with two mutual negative feedback motifs, which are controlled by a single morphogen (denoted by M) independently. (B) Multiple thresholds in a morphogen gradient. If the two mutual negative feedback motifs have different threshold values, the responding tissue is divided into three compartments (differently coloured) with two sharp boundaries. These compartments express different combinations of genes according to their position in the morphogen gradient. In theory, any number of mutual negative feedback motifs can be incorporated in the scheme, thereby generating multiple thresholds. Click here for file
