# Supplementary figures and images for: A mechanism for the sharp transition of morphogen gradient interpretation in Xenopus
Source: BMC Dev Biol. 2007 May 16;7:47. doi: 10.1186/1471-213X-7-47 (PMC1885807; doi:10.1186/1471-213X-7-47)

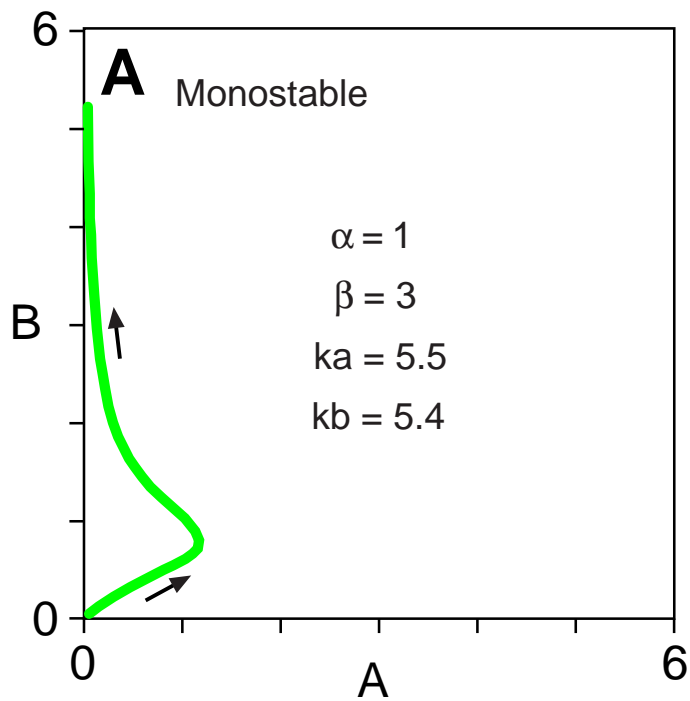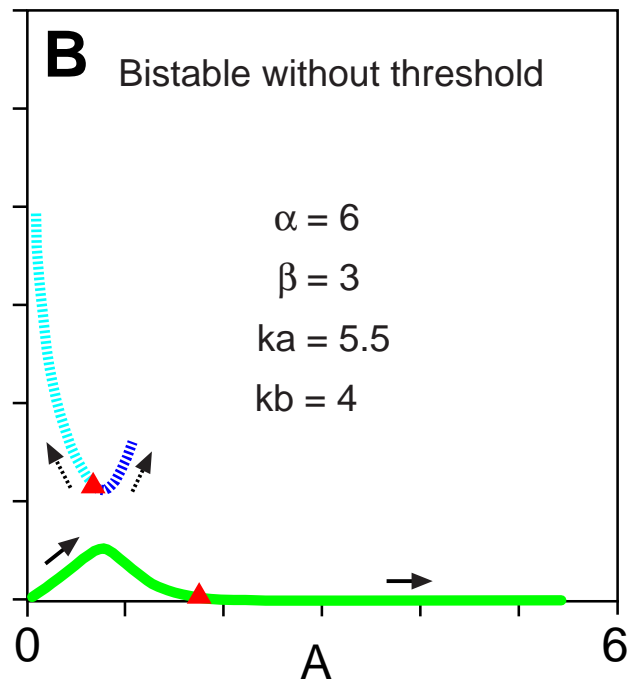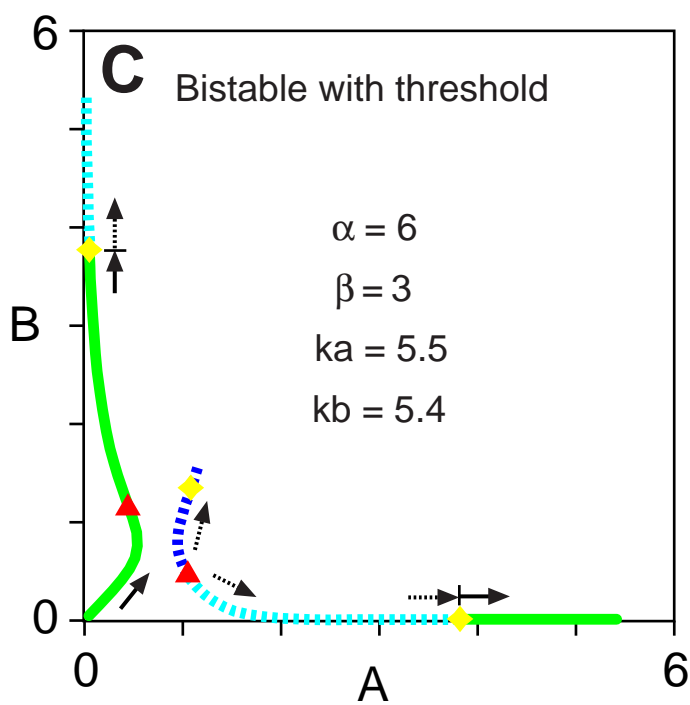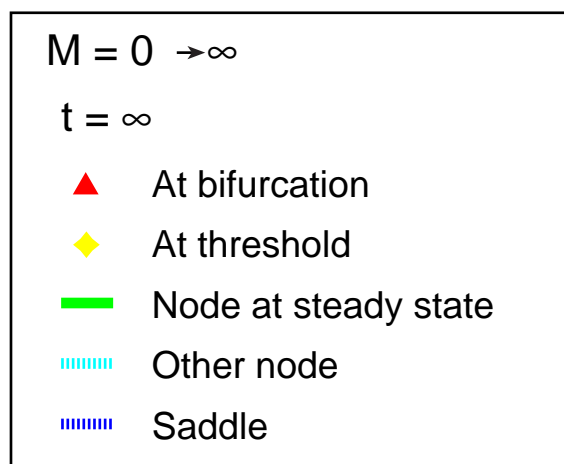

Supplement: Additional File 1 — Three possible behaviours for the system described in Figure 1A: monostable, bistable without a threshold, and bistable with a threshold. This system has two variables (A and B) that change over time according to equations (1) and (2) in the main text. The state of the system at a given time point is defined by the values (A, B) and can be plotted as a single point (i.e. phase point) on the two-dimensional phase plane illustrated in Fig. 1B. The phase point moves around the phase space with a speed defined by the equations (1) and (2) in the main text. This system like others with a mutual negative feedback motif undergoes a saddle-node bifurcation (Kobayashi H et al (2004) Proc Natl Acad Sci USA 101: 8414–8419). Nodes and saddles are the points on the phase plane where the speed of the phase point is zero, and therefore derive from the solution of a coupled equation where the left hand sides of equations (1) and (2) are replaced with 0. In our system, there are three fixed points when bifurcation occurs, two of which are stable nodes and the other is an unstable saddle point. A saddle point is analogous to a pointed summit where a ball never rests. At steady state, the phase point rests at one of the two stable nodes (see Fig. 1B). The figure shows how the positions of these nodes and the saddle change in the phase plane (A, B) as M increases from 0 to 8 as indicated by the arrows. Green curves indicate the nodes where the phase point reaches at steady state after starting from (0, 0). (A) Monostable. There is just one node irrespective of the value of M. (B) Bistable without threshold. When M is small there is only one node. As M increases, bifurcation occurs (red triangle) and another node appears, but the phase point always ends up at the original node at steady state. (C) Bistable with a threshold. When M reaches the threshold (yellow diamond), the position of the phase point at steady state shifts from the original node to the other node created at the bifurca [file 1471-213X-7-47-S1.pdf]

**A**

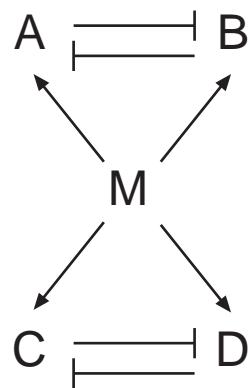

**B**

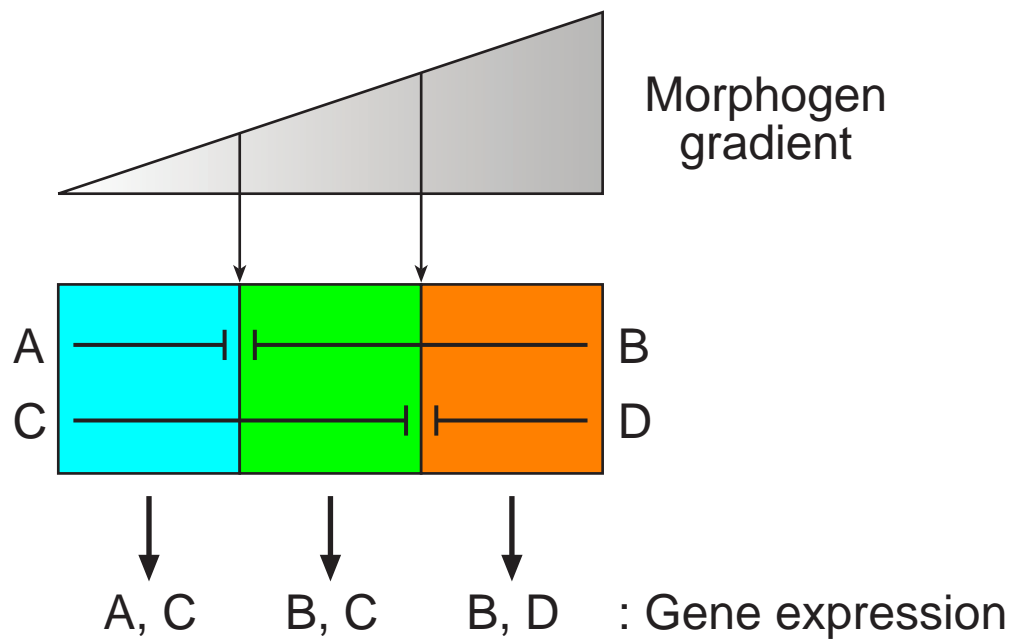

Supplement: Additional File 2 — Creation of multiple thresholds by combination of mutual negative feedback motifs. (A) A network structure with two mutual negative feedback motifs, which are controlled by a single morphogen (denoted by M) independently. (B) Multiple thresholds in a morphogen gradient. If the two mutual negative feedback motifs have different threshold values, the responding tissue is divided into three compartments (differently coloured) with two sharp boundaries. These compartments express different combinations of genes according to their position in the morphogen gradient. In theory, any number of mutual negative feedback motifs can be incorporated in the scheme, thereby generating multiple thresholds. [file 1471-213X-7-47-S2.pdf]
